# Comparative transcriptome analysis provides key insights into seedling development in switchgrass (*Panicum virgatum* L.)

**DOI:** 10.1186/s13068-019-1534-4

**Published:** 2019-08-05

**Authors:** Shumeng Zhang, Fengli Sun, Weiwei Wang, Guoyu Yang, Chao Zhang, Yongfeng Wang, Shudong Liu, Yajun Xi

**Affiliations:** State Key Laboratory of Crop Stress Biology for Arid Areas, College of Agronomy, Northwest A&F University, Yangling, 712100 Shaanxi China

**Keywords:** Switchgrass, Seedling development, Transcriptome, Circadian rhythm, Gibberellic acid, Thiamine metabolism

## Abstract

**Background:**

Switchgrass (*Panicum virgatum* L.), a warm-season perennial C4 plant, can be used as a forage plant, a soil and water conservation plant, a windbreak plant, and as a good source of biofuels and alternative energy with low planting costs. However, switchgrass exhibits low rates of seedling development compared to other crops, which means it is typically out-competed by weeds. There is a large variation in seedling development rates among different plantlets in switchgrass, which limits its usefulness for large-scale cultivation. Little is currently known about the molecular reasons for slow seedling growth.

**Results:**

Characterization of the seedling development process via growth indices indicated a relatively stagnant growth stage in switchgrass. A total of 678 differentially expressed genes (DEGs) were identified from the comparison of transcriptomes from slowly developed (sd) and rapidly developed (rd) switchgrass seedlings. Gene ontology and pathway enrichment analysis showed that DEGs were enriched in diterpenoid biosynthesis, thiamine metabolism, and circadian rhythm. Transcription factor enrichment and expression analyses showed MYB-related, bHLH and NAC family genes were essential for seedling growth. The transcriptome results were consistent with those of quantitative real-time polymerase chain reaction. Then, the expression profiles of maize and switchgrass were compared during seedling leaf development. A total of 128 DEGs that play key roles in seedling growth were aligned to maize genes. Transcriptional information and physiological indices suggested that several genes involved in the circadian rhythm, thiamine metabolism, energy metabolism, gibberellic acid biosynthesis, and signal transduction played important roles in seedling development.

**Conclusions:**

The seedling development process of switchgrass was characterized, and the molecular differences between slowly developed and rapidly developed seedlings were discussed. This study provides new insights into the reasons for slow seedling development in switchgrass and will be useful for the genetic improvement of switchgrass and other crops.

**Electronic supplementary material:**

The online version of this article (10.1186/s13068-019-1534-4) contains supplementary material, which is available to authorized users.

## Background

Switchgrass, a warm-season C4 grass indigenous to North America, is regarded as a model plant for cellulosic ethanol production due to its wide adaptability, high salt tolerance, high yield, perennation, and ease of management [1, 2]. It has low requirements for natural conditions, can grow on marginal land, and obtains high yields [2]. The advantages of switchgrass have made it a great prospect for industrial applications. However, switchgrass reproduces exclusively by seeds in the field, and there can be large variations in development rates within a single batch of seedlings. Slow-growing seedlings cannot compete with weeds, seriously affecting the development of switchgrass. It is important to understand the reasons for slow seedling development to improve large-scale cultivation of switchgrass and maximize its economic benefits as a model bioenergy crop [3, 4].

Plant seedling development involves many metabolic processes that are precisely regulated by signal transduction pathways, and this regulation is influenced by environmental interactions that can lead to a variety of gene expression patterns [5–7]. The rate of seedling development is related not only to seed size and planting depth, but also to water and nutrient availability, illumination time, light intensity, and temperature [8]. In addition to these environmental factors, many other factors in the plant affect the rate of seedling development.

Circadian rhythm is a 24-h endogenous circadian clock that adapts to day/night changes [9], which can greatly influence plant photomorphogenesis and seedling development processes [10, 11]. The plant circadian rhythm system consists of three parts: input pathways, the core oscillator, and output pathways [12]. The input pathways, such as red receptor PHYs and blue receptors CRYs, transmit changes in environmental signals, such as natural light, temperature, and nutrition, to the core oscillator, which regulates the output pathways, such as physiological and biochemical processes, involved in plant growth and development [13]. The circadian clock has various components, such as *CCA1*, *LHY* (*LATE ELONGATED HYPOCOTYL*), *TOC1* (*TIMING OF CAB EXPRESSION 1*, also known as *pseudo*-*response regulator 1*, *PRR1*) and other *PRRs* (*PRR3*, *PRR5*, *PRR7*, *PRR9*), *GI* (*GIGANTEA*), *ZTL*, *CHE* (*CCA1 HIKING EXPEDITION*), *FKF1* (*FLAVIN*-*BINDING,* *KELCH* *REPEAT, F*-*BOX 1* *protein*) [13–15]. The genetic expression of important components in the circadian clock causes changes in various aspects of plant photomorphogenesis. CIRCADIAN CLOCK ASSOCIATED 1 (CCA1) and ZEITLUPE (ZTL) regulate hypocotyl elongation in *Arabidopsis thaliana* [16, 17]. Seedling growth is affected by phytochrome interacting factors (PIFs) 4 and 5, by directly controlling auxin signaling [18]. Circadian regulation of nocturnal stomatal conductance enhances carbon assimilation and growth in *Eucalyptus camaldulensis* [19]. The circadian rhythm also regulates growth vigor in hybrids and allopolyploids and influences rhizosphere community structure and function in plants [20, 21]. Additionally, it functions in conjunction with phytohormones to regulate plant metabolism [14].

Plant hormones, such as gibberellic acids (GAs), cytokinins (CKs), and brassinosteroids (BRs), participate in early plant developmental stages, and abnormalities in their synthesis or signal transduction pathways can seriously affect plant development. GAs play an important role during the early seedling growth process, including seed germination, stem elongation, and leaf growth [22–28]. Loss-of-function mutations in positive regulators of GA signaling or GA biosynthetic genes (e.g., *sly1*, *pkl*, or *ga1*) display a typical dwarf phenotype, whereas knocking out GA signaling repressors (e.g., SPY or DELLA proteins) results in GA-independent growth [29–32]. CKs and BRs together promote cell division and differentiation. Overexpression of various *Arabidopsis* response regulators (ARRs) leads to differential responses to CKs that impact seedling development [33]. Loss of function of the BR signaling components brassinosteroid-insensitive (BRI)1 and BRI1-associated receptor kinase (BAK)1 can cause plants to display reduced BR sensitivity and defective growth compared to wild type [34–36].

As plants must allocate limited resources for survival, growth, and reproduction, their life history strategies would be a trade-off between growth rate and life span [37–39]. Rapid growth is usually accompanied by high reproduction and short life span, while slow growth is accompanied by low reproduction and long life span [37]. Leaf development has the same trade-off in the investment of growth energy, and plants either produce *cheap* leaves (high specific leaf area [SLA; the ratio of leaf area to dry mass], short life span) or *expensive* leaves (low SLA, long life span) [37, 40]. Perennial grasses, having leaves that must persist for longer than those of annuals, generally produce *expensive* leaves with a low SLA, which represents the plant fixing relatively less carbon per unit weight and consuming more in respiration proportionally [41]. It was found that slow-growing plants had a greater allocation to support and defense functions, which resulted in differences in their chemical composition compared to fast-growing plants [42–46]. Perennial grass seedling development is a complex process that involves many interdependent factors, making it difficult to study thoroughly. Studies of switchgrass seedling development are limited, and most research has focused on environmental factors. Mycorrhizal fungi and broad-spectrum herbicide applications can improve the development of switchgrass seedlings [47–49]. However, few studies have investigated the molecular reasons underlying slow development of seedlings.

Parallel assays of gene expression, such as transcriptomic analyses focusing on seedling development, might improve our understanding of these complicated interactions. Next-generation sequencing has revolutionized the use of DNA sequence information in the biological sciences. In rubber tree seedlings, comparative transcriptomic analyses between two hybrids and their parents were conducted to elucidate seedling growth heterosis [50]. Transcriptome analysis of maize seedlings revealed a number of genes that were expressed early in the seedling growth process and contributed to the final leaf size and biomass generation of the plant [51]. Thus, transcriptome data can have great value in understanding how patterns of gene expression are reflected in the ultimate phenotype. In this study, the switchgrass seedling development process was characterized, and then, the transcriptomes from slowly developed (sd) and rapidly developed (rd) seedlings were sequenced and compared. The differentially expressed genes (DEGs) related to the slow development of switchgrass seedlings were identified and analyzed based on transcriptome data, annotations, and previous studies. DEGs were also compared to maize leaf development genes to further assess the effect of the genes during seedling development. Transcriptional information and physiological indices revealed that differential expression of genes involved in the circadian rhythm, thiamine metabolism, energy metabolism, GA biosynthesis, and signal transduction may underlie the distinct seedling development patterns of sd and rd seedlings.

## Methods

### Plant materials and experimental design

**Fig. 1:**
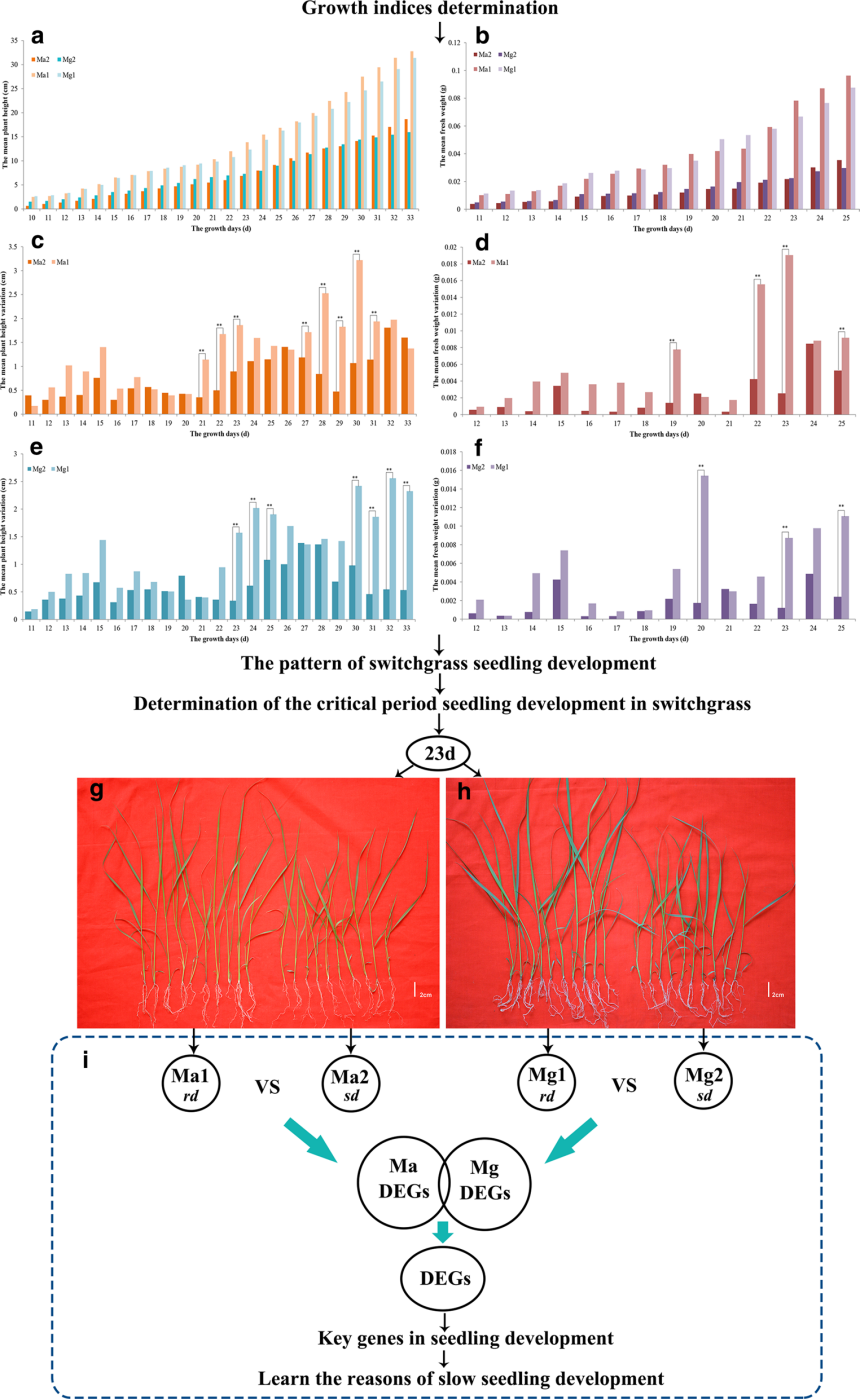
Experimental design and methods for comparing differentially expressed genes (DEGs). **a** Mean plant heights of sd and rd switchgrass seedlings. Ma1 represents the mean plant height of rd, and Ma2 represents the mean plant height of sd. Identical calculations were performed in the Mg plants. The *X*-axis shows the days after sowing (d), and the *Y*-axis shows the mean seedling height (cm). **b** Mean fresh weights of sd and rd switchgrass seedlings. Ma1 represents the mean weight of rd, and Ma2 represents the mean weight of sd. Identical calculations were performed in the Mg plants. The *X*-axis shows the number of days after sowing (d), and the *Y*-axis shows the mean seedling fresh weight (g). **c** Mean plant height variation of Ma. Two asterisks indicate significant difference at *P* < 0.01, and hereinafter. The X-axis shows the days after sowing (d), and the *Y*-axis shows the mean plant height variation (cm). **d** Mean fresh weight variation of Ma. The *X*-axis shows the number of days after sowing (d), and the *Y*-axis shows the mean seedling fresh weight variation (g). **e** Mean plant height variation of Mg. The *X*-axis shows the days after sowing (d), and the *Y*-axis shows the mean plant height variation (cm). **f** Mean fresh weight variation of Mg. The *X*-axis shows the number of days after sowing (d), and the *Y*-axis shows the mean seedling fresh weight variation (g). **g** Phenotype of Ma on day 23. Scale bar = 2 cm. **h** Phenotype of Mg on day 23. Scale bar = 2 cm. **i** Comparison methods of DEGs

The rd and sd seedlings used in this study were derived from Ma or Mg, which were two randomly selected heterozygous individual plants derived from the representative lowland cultivar of switchgrass, Alamo. The plantlets were cultured in a greenhouse at Northwest A&F University, Yangling, China, with a 14/10-h light/dark photoperiod at a constant temperature of 30 °C. We recorded the plant height and fresh weight of seedlings daily to characterize the regularity of switchgrass seedling development and determined the critical period differentiating the rd and sd seedlings. Then, we compared the transcriptomes of rd and sd seedlings during this critical period to identify the key genes involved in the seedling development of switchgrass (Fig. 1). To ensure the universality of the results, the rd and sd seedlings derived from two single plants (Ma and Mg) were measured and sequenced. Based on the functions of these genes, the reasons for the slow development of switchgrass seedlings could be determined, which could contribute to the genetic improvement of switchgrass.

### Characterization of seedling development in switchgrass

Under greenhouse conditions, switchgrass seedlings enter their characteristic elongation phase at 30–40 days after germination. The seedling height was recorded daily and measured for 24 days. The seedling fresh weight (shoots, exclusive of the roots) was measured for 15 days beginning on growth day 11. During this period, plants received sufficient regular watering and were maintained under identical growth conditions. The data analysis of seedling height and fresh weight was performed using SPSS v23.0.

### RNA sampling and extraction

RNA samples were collected on growth day 23 after the fresh weight and plant height measurements were noted. On day 23, the rd seedlings of Ma (Ma1) and Mg (Mg1) were sampled with three biological replicates (named Ma1-1, Ma1-2, and Ma1-3, and Mg1-1, Mg1-2, and Mg1-3, respectively), and the sd seedlings of Ma (Ma2) and Mg (Mg2) were also sampled (named Ma2-1, Ma2-2, and Ma2-3, and Mg2-1, Mg2-2, Mg2-3, respectively). Each of the biological replicates contained 15 plants. The 12 samples were washed with distilled water and immediately frozen in liquid nitrogen for RNA extraction.

Total RNA was extracted from the plant materials using TRIzol Reagent (Thermo-Fisher Scientific, Waltham, MA, USA) according to the manufacturer’s instructions. The concentration and purity of the total RNA were checked using a NanoDrop spectrophotometer (Thermo-Fisher Scientific) and an Agilent 2100 Bioanalyzer (Agilent Technologies, Santa Clara, CA, USA), respectively.

### RNA sequencing and de novo assembly

Twelve cDNA libraries were constructed from cDNA prepared from rd and sd seedlings using the Illumina TruSeq RNA Sample Preparation v2 kits according to the manufacturer’s instructions (Illumina Inc., San Diego, CA, USA). The libraries were sequenced on the Illumina HiSeq 2000 instrument by BGI Tech Solutions Co., Ltd. (Shenzhen, China) using paired-end sequencing technology, and the length of paired-end reads is 90.

Raw reads produced from the sequencing were scrutinized for quality in terms of total raw reads, total clean reads, Q20 percentage (proportion of nucleotides (NTs) with quality value > 20), *N* percentage (proportion of unknown NTs in clean reads), and GC percentage. Sequence reads with adaptors of unknown NTs (numbering > 5%) or low quality (reads with quality values ≤ 10), representing more than 20%, were discarded by filter_fq software, and only high-quality sequence reads were retained for assembly [52]. The Trinity software package was used to assemble the data into unigene collections from each of the 12 cDNA libraries [53]. The Trinity package combined three independent software modules (Inchworm, Chrysalis, and Butterfly) that were applied sequentially to process large volumes of RNA-seq reads.

### Bioinformatics analyses

Unigenes were annotated and analyzed to obtain DEGs for further study. Unigene annotations were performed based on a set of sequential BLAST searches to find the most descriptive annotation for each sequence using the following databases: National Center for Biotechnology Information (NCBI) non-redundant protein and NT databases, the Swiss-Prot protein database, the Kyoto Encyclopedia of Genes and Genomes (KEGG) pathway database, and the Cluster of Orthologous Groups (COG) database [54]. The Blast2GO program was used to obtain GO annotations for each unigene, and GO functional classifications were obtained from the web Gene Ontology database (WEGO) to better understand the distribution of gene functions in switchgrass seedlings at the macrolevel [55].

DESeq2 software was used to identify DEGs with a false discovery rate (FDR) ≤ 0.1 and the expression ratios (sd/rd) > 2 or (rd/sd) > 2 in the way that the batch effect was mitigated [56]. The DEG analyses were performed between Ma1 versus Ma2, and Mg1 versus Mg2. Then, DEGs between sd and rd seedlings were those that overlapped between Ma DEGs and Mg DEGs. The relative expression of unigenes was calculated using the fragments per kb per million reads (FPKM) method [57]. GO functional and KEGG pathway analyses were carried out on the screened DEGs, from which we obtained GO and KEGG functional classification annotations and functional enrichment of DEGs for further analysis. As more DEGs were enriched in specific pathways, the expression levels of other genes in those pathways were checked comprehensively and the genes with > 1.5-fold change in expression between sd and rd seedlings were also displayed in the results.

Transcription factor (TF) family analysis was performed against the Plant Transcription Factor Database (PlantTFDB; http://planttfdb.cbi.pku.edu.cn/) from Peking University using the BLASTn algorithm from NCBI with the *e*-value set to ≤ 0.00001. The homology comparison between DEGs and maize leaf development genes was performed using the BLASTn and BLASTx algorithms, with the *e*-value set to ≤ 0.00001. The correlation data between genes and leaf traits were derived from maize research [51].

### Reverse transcription and quantitative real-time polymerase chain reaction (qRT-PCR)

cDNA was obtained from total RNA using the PrimeScript™ RT reagent kit with the gDNA Eraser (RR047A; TaKaRa, Dalian, China) according to the standard protocol. The housekeeping gene, eukaryotic elongation factor 1α (*PveEF*-*1α*), was used to detect the quality of cDNA [58]. The primers were designed using Primer Premier 6 (http://www.premierbiosoft.com/primerdesign/index.html) and are listed in Additional file 1: Table S1. qRT-PCR was performed by the QuantStudio™ 3 Flex Real-Time PCR System (Thermo Scientific) using SYBR Premix Ex Taq™ II Kit (RR820A; TaKaRa) with three technical replicates. *PveEF*-*1α* was considered as the internal control to calculate the relative gene expression level using the ΔΔ*C*_T_ method.

### Physiological indices assay

According to the results of bioinformatics analysis, GA was found to play a role in the slow development of switchgrass, so the GA contents of sd and rd seedlings were determined. The seedlings sampled for GA determination were similar to those used for RNA-Seq, with the only differences being that each sample was without roots and the sampling was performed on days 16 and 24 after sowing. The names of samples were identical to those used in RNA-Seq. The levels of GA in the samples were measured by enzyme-linked immunosorbent assay at the China Agricultural University.

## Results

### Growth indices indicate a relatively stagnant seedling development stage in switchgrass

In the early stage of the experiment, we observed that switchgrass seedlings showed different seedling development rates under the same growth conditions. To study the reasons for slow seedling development, the seedling development process in switchgrass was characterized in this study. It has been proven that there is a positive correlation between the plant leaf elongation rate and fresh weight [51]. For this reason, the plant height and fresh weight variations of the sd and rd seedlings were measured to provide an index of growth rate.

From the plant height and fresh weight curves, it was clear that sd and rd seedlings grew very slowly in the early stages (Fig. 1a, b). At 16–18 days, sd seedlings were basically in a state of stagnation, especially in terms of fresh weight (Fig. 1b, d, f). To determine the significance of the growth differences between sd and rd seedlings, Duncan’s multiple range test was used to compare the variations in plant heights and fresh weights. Combined with the variations in plant heights and fresh weights, the differences between sd and rd seedlings of Ma were significant at days 22 and 23 (*P* < 0.01, Fig. 1c, d), and the differences between sd and rd seedlings of Mg were significant at days 23 and 25 (*P* < 0.01, Fig. 1e, f). Therefore, we believed that the differences in growth during the period from day 22 to day 25 contributed to the developmental differences between sd and rd seedlings.

On day 23, the variations in plant heights and fresh weights between sd and rd seedlings in Ma and Mg were all significant (*P* < 0.01), and sd and rd seedlings could be clearly distinguished (Fig. 1g, h). On this day, the mean plant height of rd was 1.5-fold higher than that of sd in Ma and Mg (Fig. 1a). The mean weight of rd was approximately threefold greater than that of sd, and the seedlings from Mg and Ma had similar growth profiles and phenotypic variations (Fig. 1a, b). This suggested that focusing on transcriptional differences between sd and rd seedlings on day 23 may provide deeper insight into the molecular networks underlying seedling development.

### Identification of DEGs related to slow seedling development

The cDNAs of the whole seedling tissues from the sd and rd seedlings were prepared and sequenced using the Illumina HiSeq 2000 to obtain a comprehensive switchgrass seedling transcriptome. In this project, 71,715,324,960 nt were generated after removing adaptors and low-quality reads. The GC content and Q20 of the 12 samples were approximately 53% and 97%, respectively. The output statistics of the sequencing are presented in Table 1. The reads were assembled using Trinity software. After clustering, 143,357 unigenes (mean length: 1174 nt) with an N50 of 1743 nt were obtained (Additional file 2: Figure S1). These results demonstrated that the Illumina sequencing data were effective and could be used in further analyses.

**Table 1:** Statistical analysis of sequencing results

Sample	Total clean nucleotides	Q20 percentage	GC percentage
Ma1-1	5,861,904,480	97.40	53.30
Ma1-2	6,193,264,680	97.41	54.12
Ma1-3	6,073,250,400	97.35	53.89
Ma2-1	5,999,384,700	97.40	54.02
Ma2-2	5,779,720,800	96.59	52.58
Ma2-3	6,041,980,080	97.33	53.56
Mg1-1	5,759,339,580	97.33	53.21
Mg1-2	5,757,629,400	97.35	54.24
Mg1-3	5,926,153,860	96.67	52.41
Mg2-1	6,108,051,060	97.14	56.83
Mg2-2	6,073,246,800	97.31	54.89
Mg2-3	6,141,399,120	96.02	53.52

To investigate the genes involved in the seedling development rate, we used DESeq2 software to identify the genes that were differentially expressed between the seedling classes that displayed rapid or slow development. DEGs were selected from Ma2 versus Ma1, and Mg2 versus Mg1, which were equivalent to the comparison of sd and rd, respectively, from two single plants (Fig. 1i). This selection method identified 678 genes with at least a twofold difference in their expression between the sd and rd seedlings. The sd seedlings had 423 upregulated and 255 downregulated genes compared to the rd seedlings. One or more of these DEGs may be responsible for the different rates of seedling development.

### Annotation and enrichment analysis of all unigenes and DEGs

Unigenes assembled from the sequence reads were used to search the NR, Swiss-Prot, KEGG, and COG (*e*-value < 0.00001) databases, using BLASTx. The NT was queried using BLASTn (*e*-value < 0.00001). Proteins having the highest sequence similarity with the obtained unigenes were annotated using these databases (Additional file 3: Table S2). The e-value and similarity distribution of the NR annotations showed that the annotation results were credible (Additional file 4: Figure S2). The species showing the highest homology with switchgrass were *Setaria italica* (69.3%), *Sorghum bicolor* (10.5%), and *Zea mays* (8.0%) (Additional file 4: Figure S2).

**Fig. 2:**
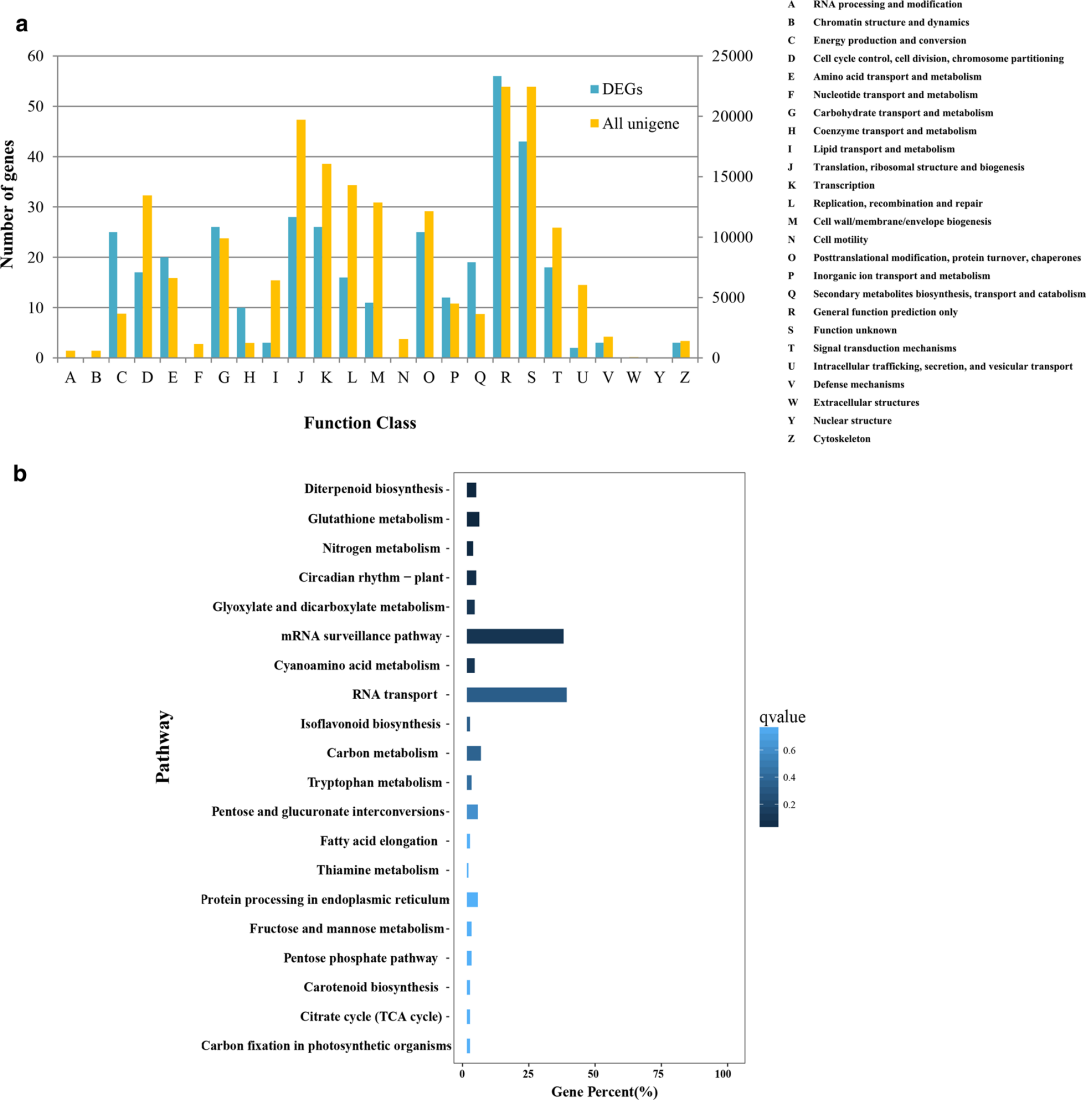
Functional classification of all unigenes and DEGs using COG and KEGG analysis. **a** Functional classification of all unigenes and DEGs using COG analysis. The horizontal coordinates are the COG functional classes. The left vertical coordinate represents the numbers of DEGs in one class, and the right vertical coordinate represents the numbers of unigenes in one class. The notations on the right list the full names of the functions in the *X*-axis. **b** Kyoto Encyclopedia of Genes and Genomes (KEGG) classification of DEGs. The vertical coordinates are the KEGG pathway functional classes. The horizontal coordinates represent the number of gene percent (%) in one class

To further understand which genes may be related to seedling development, the COG, GO, and KEGG analyses of the unigenes and DEGs were compared. Output from the COG analysis showed that, compared to the unigenes, the DEGs were enriched in genes involved in general function prediction only (R); energy production and conversion (C); and secondary metabolites biosynthesis, transport, and catabolism (Q) (Fig. 2a). The GO enrichment analysis showed that more DEGs were enriched in response to temperature stimulus, oxidoreductase activity, and intracellular organelle (Additional file 5: Figure S3). The KEGG functional annotations revealed that the DEGs were enriched in genes involved in diterpenoid biosynthesis, glutathione metabolism, nitrogen metabolism, and circadian rhythm (Fig. 2b). These data provide an initial framework for screening DEGs of seedlings with rapid or slow development rates.

### Functional analysis of specific DEGs related to seedling development

#### TF analysis

**Fig. 3:**
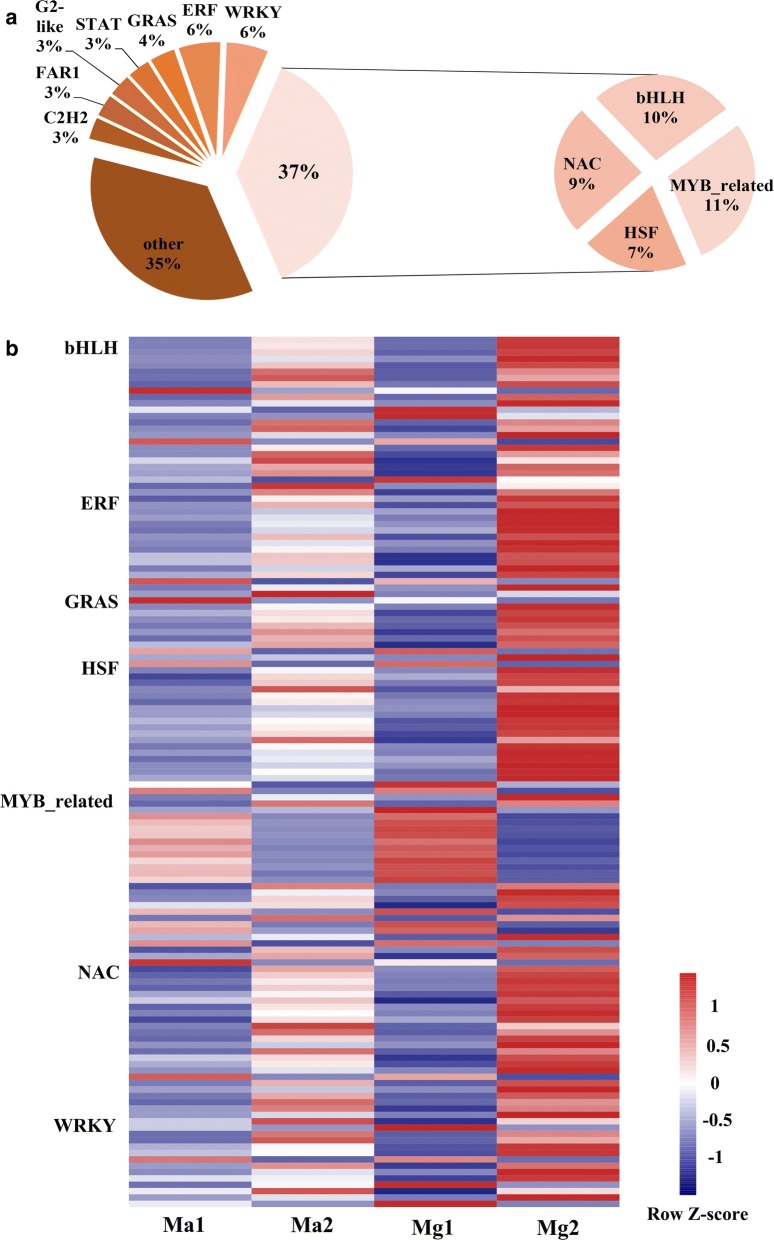
Distribution and expression of the transcription factor families of DEGs. **a** Distribution of the transcription factor families of DEGs. **b** Gene expression of the transcription factor families of DEGs. The expression data are the FPKM values of the samples centralized by Z-score transformation

Gene expression is often regulated at the transcriptional level. Therefore, the collection of DEGs was used to query the PlantTFDB from Peking University to identify TFs that might have a vital role in seedling development in switchgrass. In total, 260 DEGs were assigned to 37 TF families. Among them, MYB-related, basic helix-loop-helix (bHLH), NAC, and heat shock transcription factor (HSF) TF families were the most commonly cited (Fig. 3a). The expression levels of the most commonly cited TFs were analyzed and are displayed in Fig. 3b; they may play an important role in seedling growth and development.

#### DEGs in the GA pathway

**Fig. 4:**
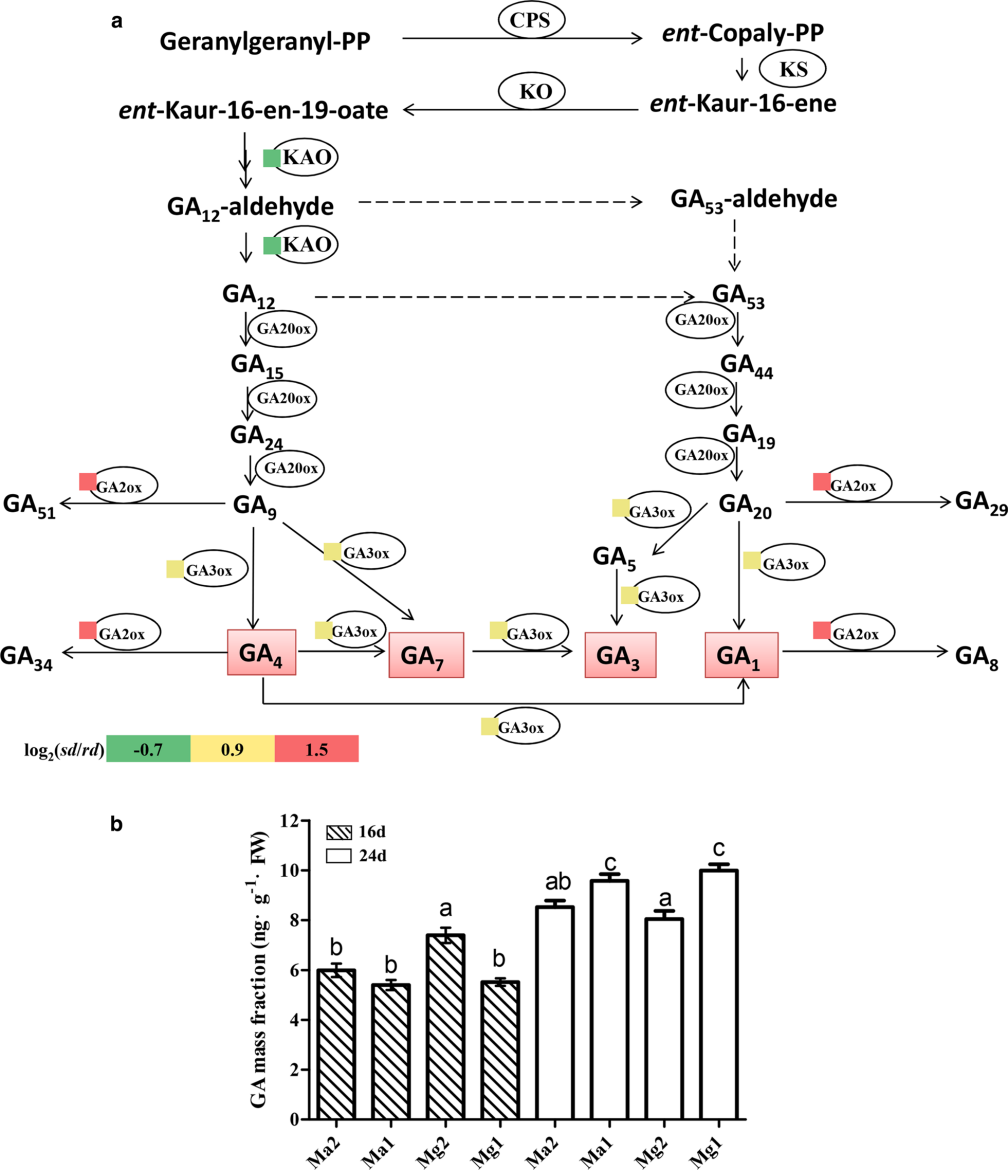
Expression of genes in the GA pathway and GA levels of sd and rd seedlings. **a** Red color indicates upregulated expression, and green indicates downregulated expression. Log_2_ (sd/rd) represents the gene fold change, where sd/rd refers to the ratio of FPKM reads. **b** Different lowercase letters indicate significant difference at *P* < 0.05

The regulation of GAs is critical for seedling development, and several bioactive GAs (such as GA_1_, GA_3_, GA_4_, and GA_7_) function in plants [59–63]. *Ent*-copalyl diphosphate synthase (CPS), *ent*-kaurene synthase (KS), *ent*-kaurene oxidase (KO), *ent*-kaurenoic acid hydroxylase (KAO), GA20-oxidases (GA20ox), and GA3-oxidases (GA3ox) are the key enzymes in the GA biosynthetic pathway; GA2ox changes bioactive GAs (GA_1_, GA_4_) to inactive GAs [63–65]. These enzymes maintain GA homeostasis and maintain normal growth in plants [62]. Our results revealed that the DEGs enriched in the diterpenoid biosynthesis pathway are enzymes related to GAs (Fig. 4a). The results indicated that, compared to rd seedlings, the sd seedlings showed upregulation of *GA2ox* (Unigene510_All, CL20643.Contig1_All) and *GA3ox* (Unigene32915_All). The expression of *KAO* (Unigene41554_All) in rd seedlings was 1.7-fold higher than in sd seedlings. In the GA signaling pathway, *scarecrow*-*like protein 3* (*SCL3,* Unigene44355_All) was upregulated in sd seedlings, and the expression levels of GA receptors *GID1* (Unigene10306_All) and *DELLA* (Unigene38958_All) in sd seedlings were both more than 1.5-fold higher than in rd seedlings (Additional file 6: Table S3). Therefore, we speculated that unusual GA levels contributed to the slow development in sd seedlings.

To confirm this speculation, we measured the GA content in the two types of seedling from Ma and Mg. The results showed that the GA levels in rd and sd seedlings had little difference in the early growth stage (day 16), but during transcriptome sampling the GA levels in rd were higher than those in sd, and the differences were significant (Fig. 4b). A correlation analysis between GA content and plant heights indicated that their correlation was significant at the 0.05 level (Additional file 7: Table S4). Therefore, GA had a large influence on seedling development in switchgrass.

#### DEGs related to circadian rhythm

**Fig. 5:**
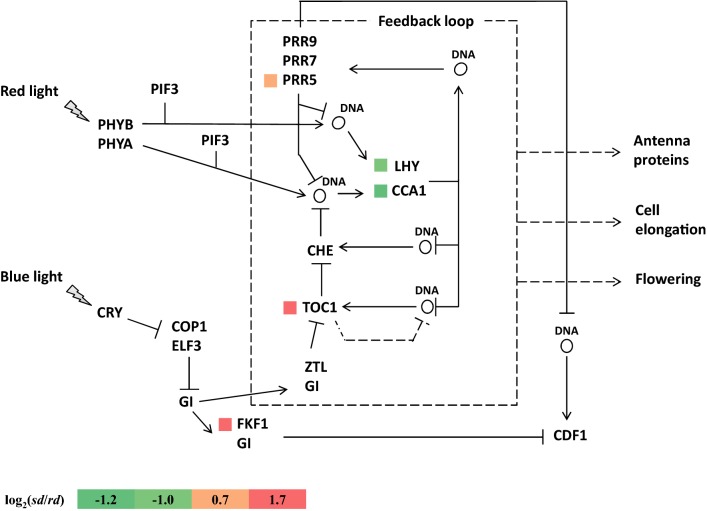
Gene expression levels in the circadian rhythm pathway. Red color indicates upregulated expression, and green indicates downregulated expression. Log_2_ (sd/rd) represents the gene fold change, where sd/rd refers to the ratio of fragments per kb per million (FPKM) reads

Within the negative feedback regulation network of plant circadian rhythm, *CCA1* and *LHY* directly repress the transcription of *TOC1*, while *TOC1* participates in *CCA1* and *LHY* activation at dawn [66, 67]. TOC1 and PRR5 proteins are targets of FKF1 for degradation [68]. In addition, CCA1 and LHY are two homologous MYB-domain transcription factors that partially overlap in function [69]. *TOC1*-*ox* of *Arabidopsis* was found to have a slow cell cycle and displayed a dwarf phenotype, with reduced plant size and small leaves [70]. Overexpression of *PRR5* repressed *LHY* promoter activity and affected its transcriptional activity [68]. Overexpression of *CCA1*, which controls the stomatal aperture and inhibits leaf senescence, enhanced leaf area and biomass of the plants compared to the wild type [71, 72]. In this study, TF analysis indicated that many DEGs belonged to the MYB-related family. Additionally, the enrichment analysis revealed that DEGs were more abundant in the rhythmic process pathway than the unigenes, and these genes were key players in the circadian clock. *FKF1* (Cl3379.contig2_All, CL17216.contig1_All, CL17216.contig2_All) was upregulated, whereas *CCA1* (CL4612.Contig2_All, CL4612.Contig5_All) and *LHY* (CL4612.Contig10_All, CL4612.Contig3_All) were downregulated in sd seedlings. The expression levels of *TOC1* (CL5358.Contig3_All) and *PRR5* (CL8053.Contig6_All) in sd seedlings were more than 1.5-fold higher than in rd seedlings (Fig. 5, Additional file 6: Table S3). The results of qPCR analysis of these genes in sd and rd seedlings were consistent with the transcriptome analysis (Fig. 7b). The different expression levels of these genes in sd seedlings may be one factor that led to their slow development; therefore, it is speculated that the differences between sd and rd seedlings of switchgrass are related to variation in the expression of rhythmic genes, and to differences in responses to light and temperature.

#### DEGs related to thiamine metabolism

Thiamine (vitamin B_l_) is a cofactor for two enzyme complexes involved in the citric acid cycle, pyruvate dehydrogenase and α-ketoglutarate dehydrogenase, and is an essential constituent of all cells and can be synthesized by the plant [73]. It contains two parts: 4-amino-5-hydroxymethyl-2-methylpyrimidine phosphate (pyrimidine) and 4-methyl-5-(2-hydroxyethyl)-thiazole phosphate (thiazole) [74–76]. Pyrimidine synthesis requires the product of *THIAMIN C* (*THIC*), and thiazole synthesis in *A. thaliana* and *Z. mays* is performed using a single enzyme, thiamine thiazole synthase (THI1) [73, 74, 77]. *THI1* was found to promote nodule growth and seed maturation [78]. *COG0212* (5-formyltetrahydrofolate cyclo-ligase-like protein) was also found to have an unknown but crucial role in thiamine metabolism [79]. In this study, enrichment analysis revealed that a greater number of genes were enriched in the thiamine metabolism pathway; moreover, *THI1* (CL9070.Contig1_All, CL9070.Contig2_All, CL9070.Contig3_All, CL9070.Contig4_All) and *THIC* (CL4091.Contig1_All, CL4091.Contig4_All) expression was upregulated in sd seedlings. The expression of *COG0212* (CL10550.Contig2_All) in sd seedlings was more than 1.5-fold higher than in rd seedlings (Additional file 6: Table S3). Differences between sd and rd seedlings may be partially caused by differences in thiamine metabolism.

#### DEGs related to energy metabolism

It has been suggested that slow-growing plants show a preference for investment in support and defense functions, which results in differences in their chemical composition compared to fast-growing plants [42–45]. They allocate more energy to the formation of cell wall components [44–46]. These plants would be expected to display low leaf area and low rates of photosynthesis and respiration. Shikimate *O*-hydroxycinnamoyltransferase (HCT, [EC: 2.3.1.133]), a key enzyme in lignin synthesis, is a major factor promoting the growth of huge stems in *Dendrocalamus sinicus*, giant bamboo, one of the largest bamboo species in the world [80]. HCT functions in the biosynthetic pathway of lignin, a major component of secondary cell walls [81]. FEI1, LRX5, and phosphomannomutase (PMM, [EC: 5.4.2.8]) are both important for cell wall function in plants [82–88], and TBL34 is essential for normal secondary wall deposition and plant growth [89]. SCL32 is related to leaf vein development in *Arabidopsis* [90]. In this study, *HCT* (Unigene47957_All), *FEI1* (CL17044.Contig1_All), *LRX5* (CL14544.Contig1_All, CL20761.Contig1_All, CL5207.Contig2_All), and *PMM* (CL6762.Contig1_All, CL6762.Contig2_All) were upregulated in sd seedlings. The expression of *TBL34* (CL3734.Contig13_All) and *SCL32* (Unigene39241_All) in sd seedlings was more than 1.5-fold higher than in rd seedlings (Additional file 7: Table S4). Many cell wall structural proteins were identified as DEGs, and they were all upregulated in sd seedlings.

Plant photomorphogenesis along with chloroplast biosynthesis is important processes for seedling development [10, 11, 91]. Early light-induced proteins (ELIPs), as nuclear-encoded thylakoid proteins in chloroplasts, are transiently induced in the very early stages of the greening process in etiolated pea seedlings [92, 93]. ELIPs have been reported as being associated mainly with chloroplast biogenesis during leaf greening and during environmental stress [94–96]. AKRP (ankyrin repeat domain-containing protein) is an ankyrin repeat protein that is essential for plastid differentiation and plant development, but its accumulation is harmful to chloroplasts [97]. In this study, GO enrichment analyses revealed that the DEGs were enriched in intracellular membrane-bounded organelle and chloroplast thylakoid lumen. ELIPs (Unigene40595_All, CL7413.Contig2_All, etc., Additional file 6: Table S3) were upregulated in sd seedlings, and the expression of AKRP (CL16114.Contig1_All) in sd seedlings was more than 1.5-fold higher than in rd seedlings. Therefore, differences in growth energy allocation may contribute to differences in seedling development rates between sd and rd seedlings; sd seedlings may have a preference for investment more in cell wall components, which may lead to slow development in switchgrass.

#### DEGs related to leaf development: comparison with maize

The relationship between transcriptome and phenotype is a major focus of research. Correlation analyses of the transcriptome of growing leaves with mature leaf parameters in a maize population have been performed [51]. A total of 1740 genes were obtained that correlated with the 4th leaf size traits (final leaf length, final leaf weight, final leaf area, final leaf width), shoot traits (shoot fresh weight, shoot dry weight), and timing traits (emergence of leaf four, time between sowing and reaching final length, time between sowing and reaching maximal growth rate, leaf elongation duration from a leaf of 5 mm until final length). Each gene was (anti-)correlated with at least one of the traits. As leaf development is a part of the seedling development process, we compared DEGs in switchgrass with those in maize to further explore the role of DEGs in plant development.

**Fig. 6:**
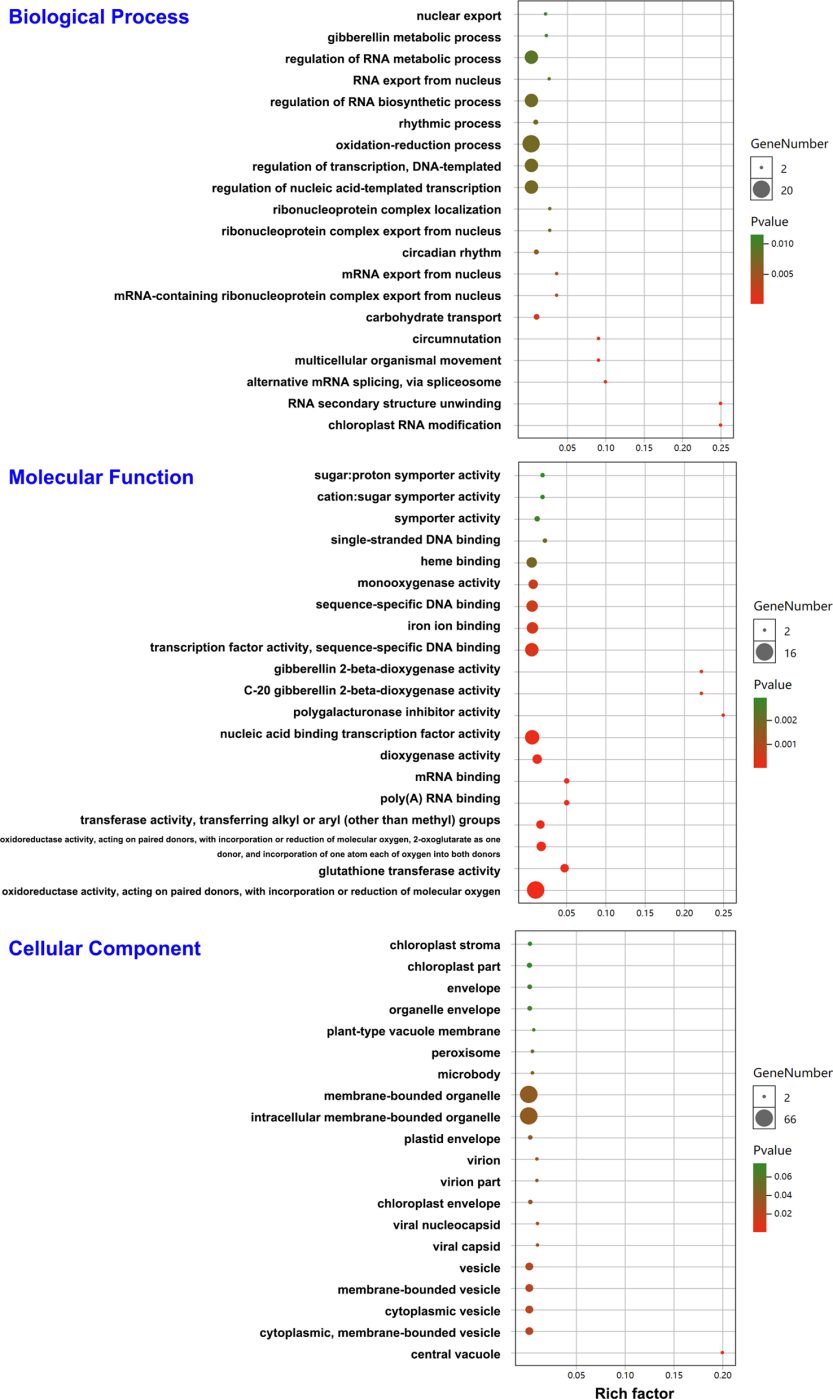
Functional classification of the DEGs homologous to maize using GO analysis. The horizontal axis shows the enrichment factors of the GO terms. The vertical axis shows the top 20 enriched GO terms in the biological process, molecular function, and cellular component categories. The size of the circle indicates the number of genes enriched in each term. The color of the circle indicates the *P* value of the enriched term

We used the BLASTn and BLASTx algorithms for homologous analysis, and 128 DEGs were aligned to the maize genes. Approximately 70% of the aligned genes upregulated in sd seedlings were negatively correlated with leaf traits in the maize study (Additional file 8: Figure S4). The expression of these genes may result in the slow development of sd seedlings. The functions of the aligned DEGs were analyzed by annotations and GO enrichment analysis. The GO enrichment analysis revealed that DEGs were enriched more in the oxidation–reduction process, gibberellin metabolic process, transcription factor activity, chloroplast part, central vacuole, and vesicle terms (Fig. 6), indicating that sd and rd seedlings differed in chloroplast development, photosynthesis, and gibberellin metabolic processes (Additional file 9: Table S5). The common effects of them led to the different development rates in sd and rd seedlings.

### Verification of DEGs by qRT-PCR

**Fig. 7:**
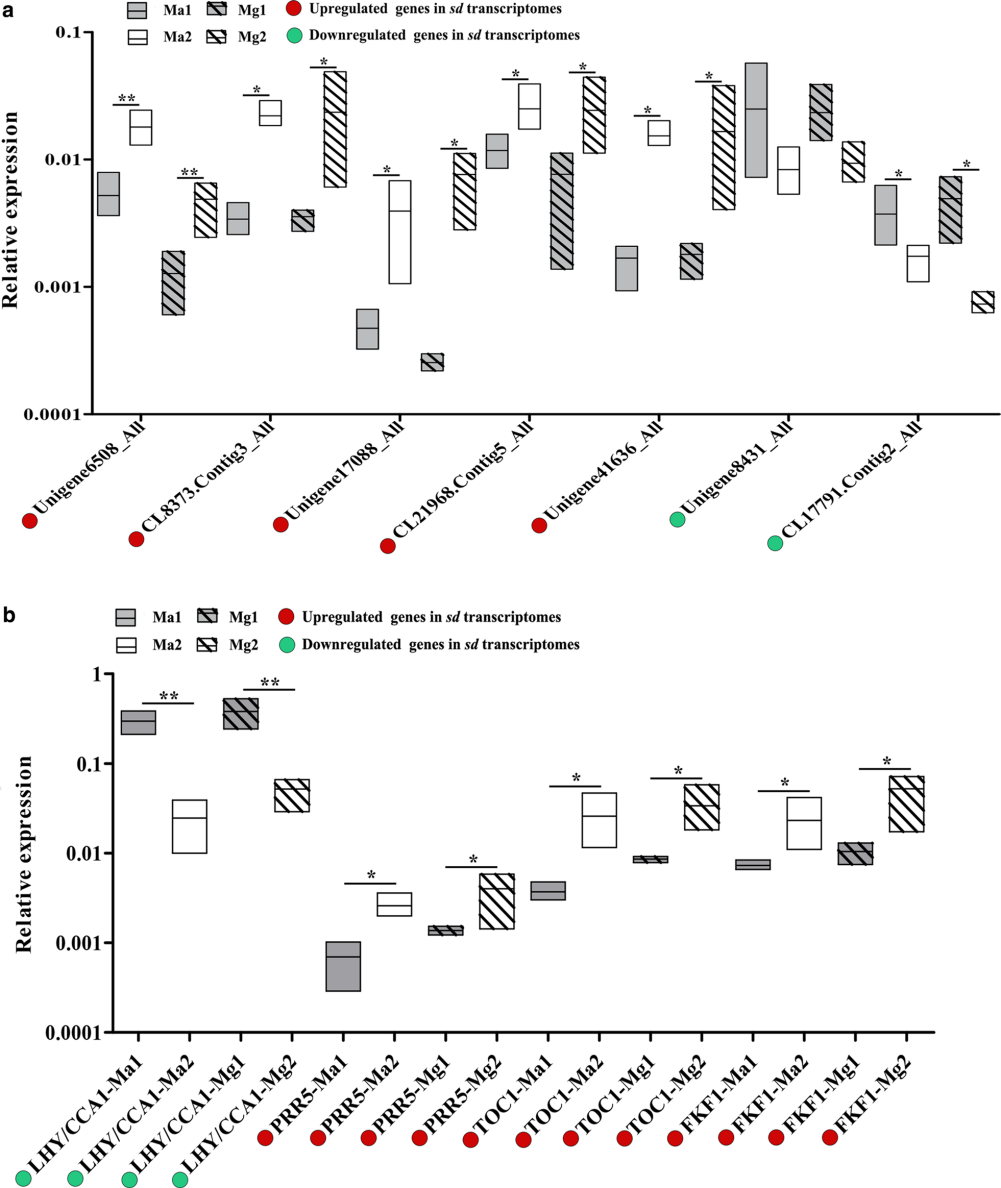
Boxplot of DEG verification by quantitative real-time polymerase chain reaction (qRT-PCR). **a** Boxplot of randomly selected DEG verification by qRT-PCR. **b** Boxplot of DEG expressions in the circadian rhythm pathway by qRT-PCR. An asterisk indicates significant difference at *P* < 0.05; two asterisks indicate significant difference at *P* < 0.01

The expression levels of the DEGs randomly selected from the transcriptome as well as those related to circadian rhythm in the functional analysis were detected by qRT-PCR experiments to validate the transcriptome data. The selected genes were Unigene6508_All, CL8373.Contig3_All, Unigene17088_All, CL21968.Contig5_All, Unigene41636_All, Unigene8431_All, and CL17791.Contig2_All. The results showed that the expression levels of Unigene6508_All, CL8373.Contig3_All, Unigene17088_All, CL21968.Contig5_All, Unigene41636_All, and CL17791.Contig2_All were significantly different in sd and rd seedlings (*P* < 0.05, Unigene6508_All: *P* < 0.01, Fig. 7a), and the expression levels of DEGs related to circadian rhythm were also significantly different in sd and rd seedlings (*P* < 0.05, LHY/CCA1: *P* < 0.01, Fig. 7b). Therefore, the qRT-PCR results were in agreement with the transcriptome data.

## Discussion

In the field, switchgrass seedlings have an inconsistent seedling development process under the same growth conditions. This difference in seedling development rate led to the seedlings being out-competed by weeds and seriously affected the large-scale plantation and promotion of switchgrass. To understand the reasons underlying slow seedling development, the seedling development process was characterized in switchgrass; then, rapidly and slowly developed seedlings were compared in terms of their gene expression profiles to identify DEGs between the two classes of seedlings.

Overexpression of *GA2ox* can significantly decrease the GA level in plants [64, 98]. Wuddineh et al. identified *GA2ox* in switchgrass [99] and found that overexpression of *PvGA2ox5* and *PvGA2ox9* in plants decreased the GA level and reduced shoot growth. They also found that *PvGA2ox6* had the same expression profiles as *PvGA2ox9*. The DEGs annotated as *GA2ox* in our study were aligned to the results of the above-mentioned study and were identified as *PvGA2ox6b* and *PvGA2ox9b* (Table 2). In sd seedlings, the changes in gene expression in the GA biosynthetic pathway may lead to their lower GA level; the physiological data of GA also indicated this result.

**Table 2:** Upregulation of *PvGA2ox* in sd seedlings

GeneID	Wuddineh et al.	PvCDS v4.1	PvCDS v1.1	Log_2_ (sd/rd)
Unigene510_All	PvGA2ox9b	Pavir.1NG349400.1	Pavir.J11300	1.4
CL20643.Contig3_All	PvGA2ox6b	Pavir.7KG234700.1	Pavir.Ga01287	1.3
CL20643.Contig1_All	PvGA2ox6b	Pavir.7NG327800.1	Pavir.Gb01218	1.8

The roles of GAs are complex; GA levels can not only directly affect seedling growth, but also have an indirect effect on seedling development through circadian rhythm and mycorrhizal formation via signal transduction. SCARECROW-LIKE 3 (SCL3) and DELLA are involved in the GA signal transduction pathway [100, 101]. *GA2ox* and *SCL3* are upregulated in *Lotus japonicas* infected with arbuscular mycorrhizas [102]. DELLA also plays an important role in mycorrhizal signaling pathways [103] and mediates *CCA1* in the interaction between GAs and the circadian clock, to regulate hypocotyl elongation in *A. thaliana* [16]. In our study, *GA2ox* and *SCL3* were upregulated in sd seedlings; *CCA1* and *DELLA* were both differently expressed. Therefore, GA affects seedling development in several ways, resulting in differences in switchgrass seedling development rates.

Thiamine metabolism can affect plant growth in various ways and plays a fundamental role in glycolysis, the pentose phosphate pathway, and the tricarboxylic acid cycle as an enzymatic cofactor [73, 75]. Meanwhile, *THI1* and *THIC* showed a response to various abiotic stresses and may increase tolerance to mitochondrial DNA damage [104]; furthermore, there was an association between thiamine metabolism and two symbioses, root nodule symbiosis and arbuscular mycorrhiza [105, 106]. In *L. japonicus*, rhizobium inoculation resulted in enhanced expression of *THI1* and *THIC*. In the *thic* mutant of *Lotus corniculatus*, the content of thiamine decreased in roots and increased in arbuscular mycorrhizal fungi (AMF) [78, 105]. Low thiamine levels rendered spores of AMF less viable [105]. In this study, *THIC* and *THI1* were upregulated in sd seedlings, where this upregulation may result from stress or the growth of rhizosphere microorganisms. The physiological indices, including malondialdehyde, H_2_O_2_, superoxide anion, glutathione, and ascorbic acid, which can reflect the extent of environmental stress to plants, were measured in sd and rd seedlings, and no significant differences were found [107]. Therefore, the upregulation of *THI1* and *THIC* in sd seedlings was not caused by abiotic stress and may have been due to infection with rhizosphere microorganisms. The gene *MLO1* (Unigene19129_All) related to mycorrhiza formation was found to be upregulated in sd seedlings [102]. The different expression levels of mycorrhizal response genes between sd and rd seedlings indicated that there were differences in mycorrhiza formation and this may lead to differences in sd and rd seedling development rates.

## Conclusions

Switchgrass can be used for livestock, water and soil conservation, and in the bioenergy sector, given the high water and fertilizer use efficiency of adult plants and its strong ability to adapt to adverse environmental conditions [47, 108]. Since slow and inconsistent seedling development affects large-scale plantations and reduces switchgrass utilization as a bioenergetic crop, the seedling development process was characterized in this study, and the transcriptomes of rapidly and slowly growing seedlings were compared to identify key genes involved in seedling development. Growth indices indicated a relatively stagnant seedling development stage in switchgrass, and 678 genes were identified as being involved in seedling development. Homologous analysis of switchgrass and maize identified 128 key genes related to seedling leaf development. Transcriptome analysis and physiological indices suggested that the seedling development process was influenced by differential expression of genes involved in the circadian rhythm, thiamine metabolism, energy metabolism, GA biosynthesis, and signal transduction. This study contributes to a better understanding of the regulatory reasons underpinning seedling development and growth rates. Identification of the genes directly responsible for rapid and reliable seedling development will make possible the creation of switchgrass cultivars that have more desirable growth characteristics.

## Additional files

**Additional file 1: Table S1.** Primers used in qRT-PCR.

**Additional file 2: Figure S1.** Length distribution of unigenes in switchgrass seedlings. The horizontal coordinates represent the unigene lengths, and the vertical coordinates indicate the numbers of unigenes.

**Additional file 3: Table S2.** Summary statistics of functional annotations in various databases.

**Additional file 4: Figure S2.** Species distribution of the NR annotation results. a. The e-value distribution of the NR annotation results. b. The similarity distribution of the NR annotation results. c. The species distribution of the NR annotation results.

**Additional file 5: Figure S3.** Functional classification of DEGs using GO analysis. The horizontal axis shows the enrichment factors of the GO terms. The vertical axis shows the top 20 enriched GO terms in the biological process, molecular function, and cellular component categories. The size of the circle indicates the number of genes enriched in each term. The color of the circle indicates the *P* value of the enriched term.

**Additional file 6: Table S3.** Expression of genes related to circadian rhythm, thiamine metabolism, the GA pathway, and energy metabolism.

**Additional file 7: Table S4.** The correlation of the GA content and plant heights.

**Additional file 8: Figure S4.** Homology analysis of switchgrass and maize leaf development. The left panel shows DEGs in switchgrass that are homologous to maize. Red color indicates upregulated expression, and green indicates downregulated expression in sd seedlings. The right panel shows gene IDs for maize. Orange and blue indicate positive and negative correlations, respectively, with leaf development in maize.

**Additional file 9: Table S5.** Examples of genes that were homologous to maize during leaf development in switchgrass seedlings.

## Data Availability

The raw sequence data have been deposited in the National Center for Biotechnology Information (NCBI) database (https://www.ncbi.nlm.nih.gov/sra/SRP148735). All other relevant supplementary data are provided within this manuscript as Additional files 1, 2, 3, 4, 5, 6, 7, 8, 9.

## Abbreviations

AMF: arbuscular mycorrhizal fungi
BLAST: basic local alignment search tool
BR: brassinosteroid
cDNA: complementary DNA
CK: cytokinin
COG: Cluster of Orthologous Groups
DEG: differentially expressed gene
FPKM: fragments per kilobase of exon per million fragments mapped
GA: gibberellic acid
GO: gene ontology
KEGG: Kyoto Encyclopedia of Genes and Genomes
NR: NCBI non-redundant protein sequences database
NT: nucleotide collection database
qRT-PCR: quantitative real-time PCR
rd: rapidly developed
RNA-Seq: RNA sequencing
sd: slowly developed
SLA: special leaf area
TF: transcription factor
