# Supplementary figures and images for: Comparative transcriptome analysis provides key insights into seedling development in switchgrass (Panicum virgatum L.)
Source: Biotechnol Biofuels. 2019 Aug 5;12:193. doi: 10.1186/s13068-019-1534-4 (PMC6683553; doi:10.1186/s13068-019-1534-4)

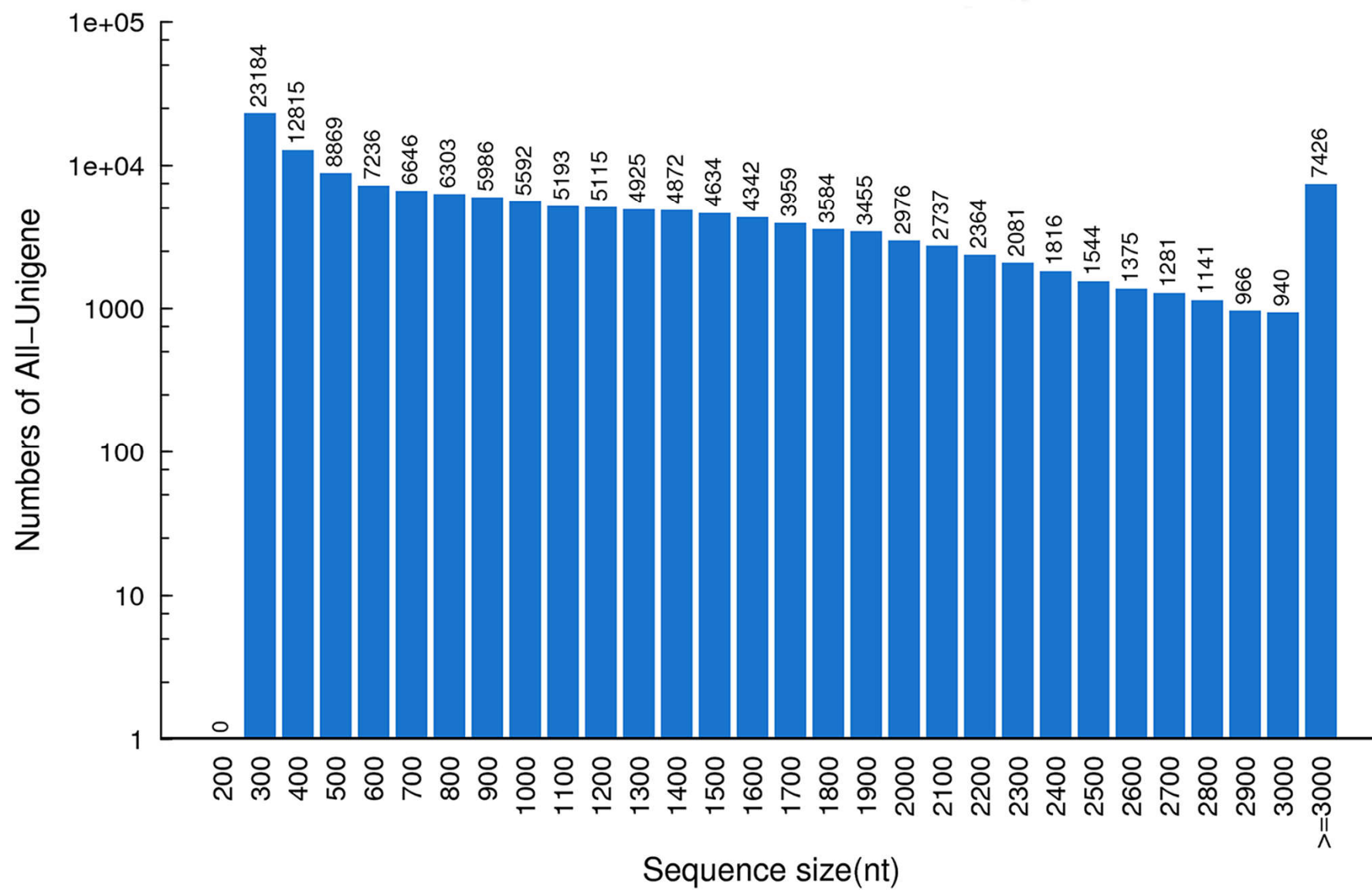

Supplement: Supplementary file 2 — Additional file 2: Figure S1. Length distribution of unigenes in switchgrass seedlings. The horizontal coordinates represent the unigene lengths, and the vertical coordinates indicate the numbers of unigenes. [file 13068_2019_1534_MOESM2_ESM.pdf]

a.

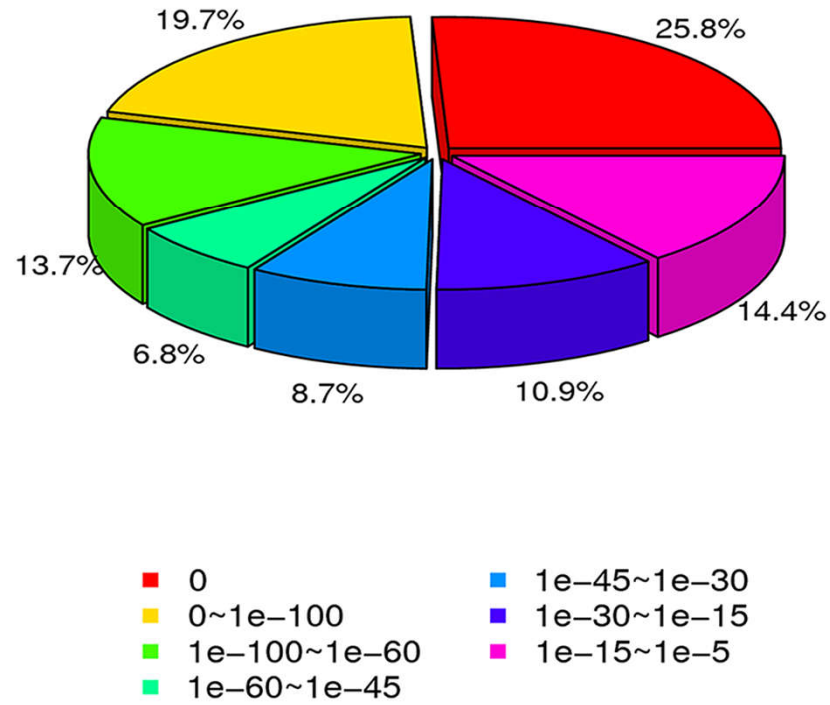

b.

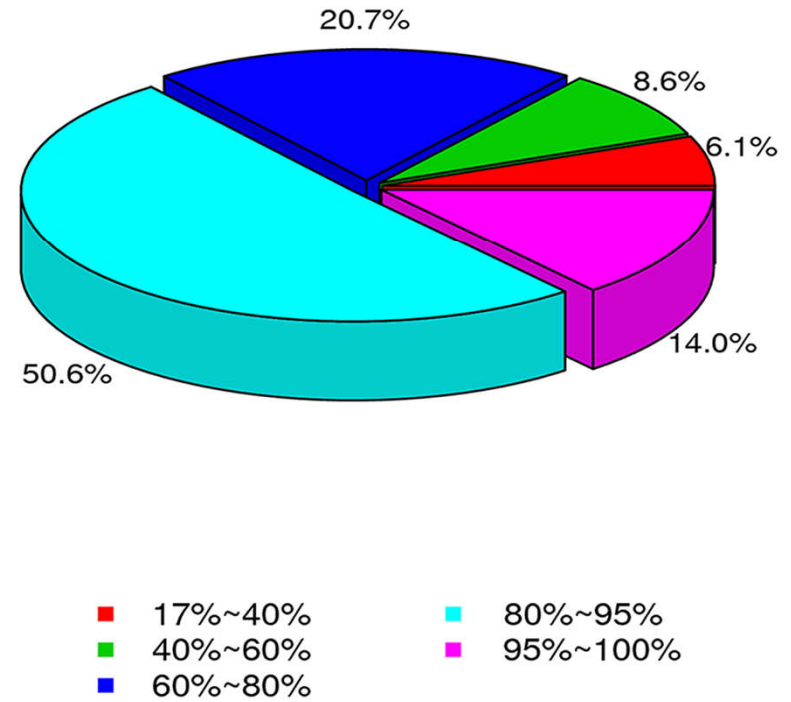

c.

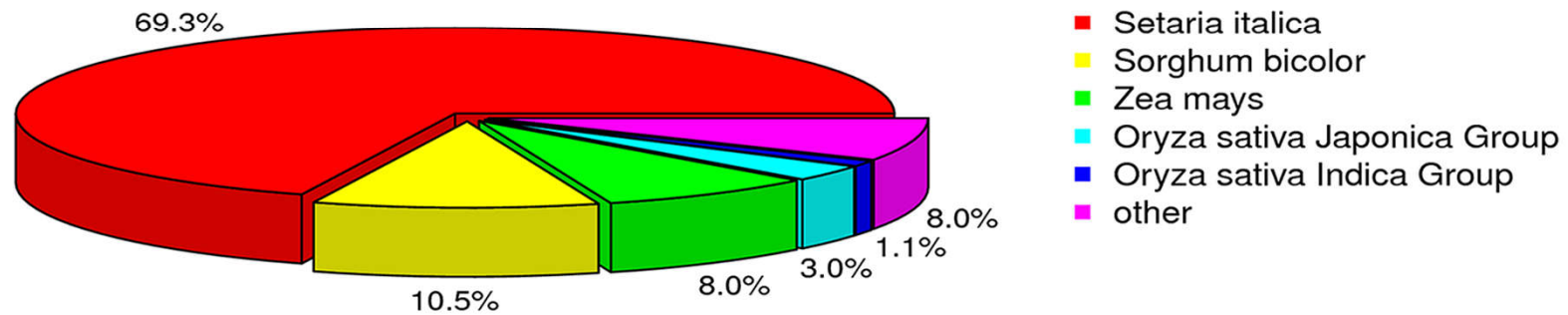

Supplement: Supplementary file 4 — Additional file 4: Figure S2. Species distribution of the NR annotation results. a. The e-value distribution of the NR annotation results. b. The similarity distribution of the NR annotation results. c. The species distribution of the NR annotation results. [file 13068_2019_1534_MOESM4_ESM.pdf]

Biological Process

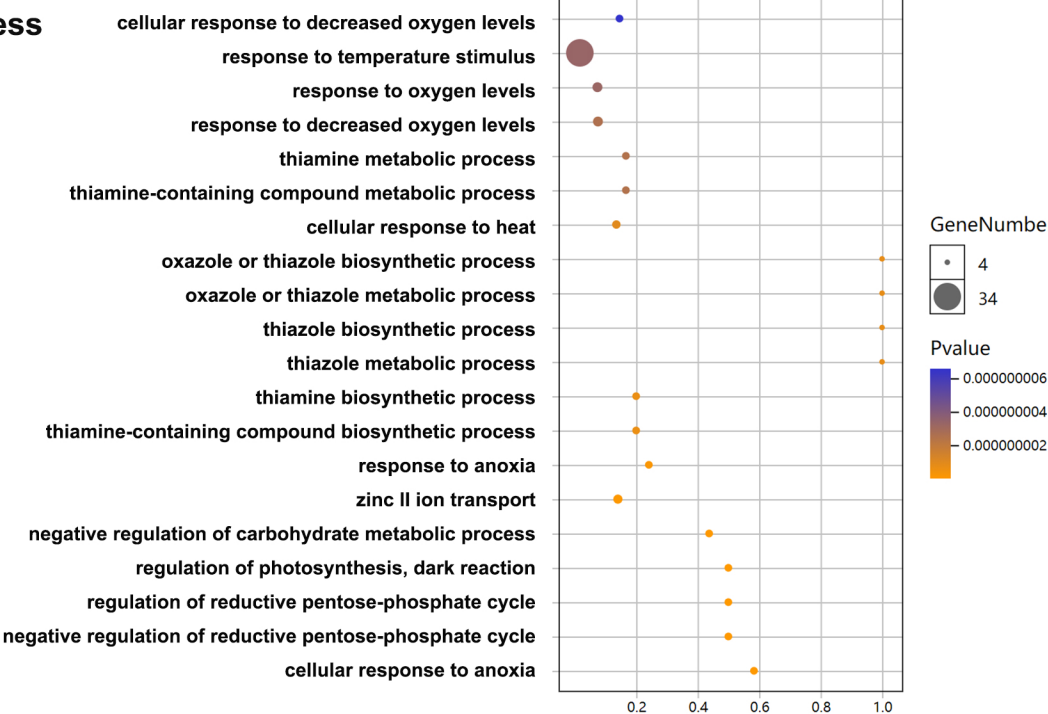

Molecular Function

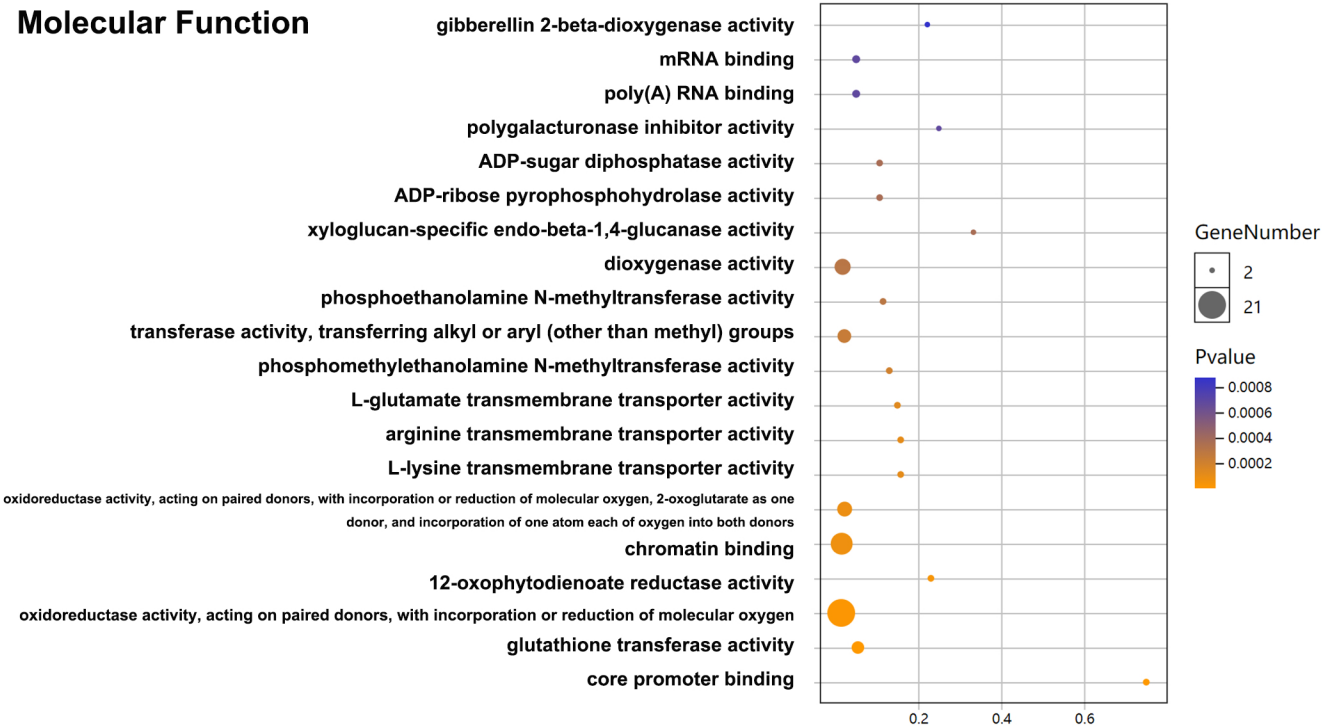

Cellular Component

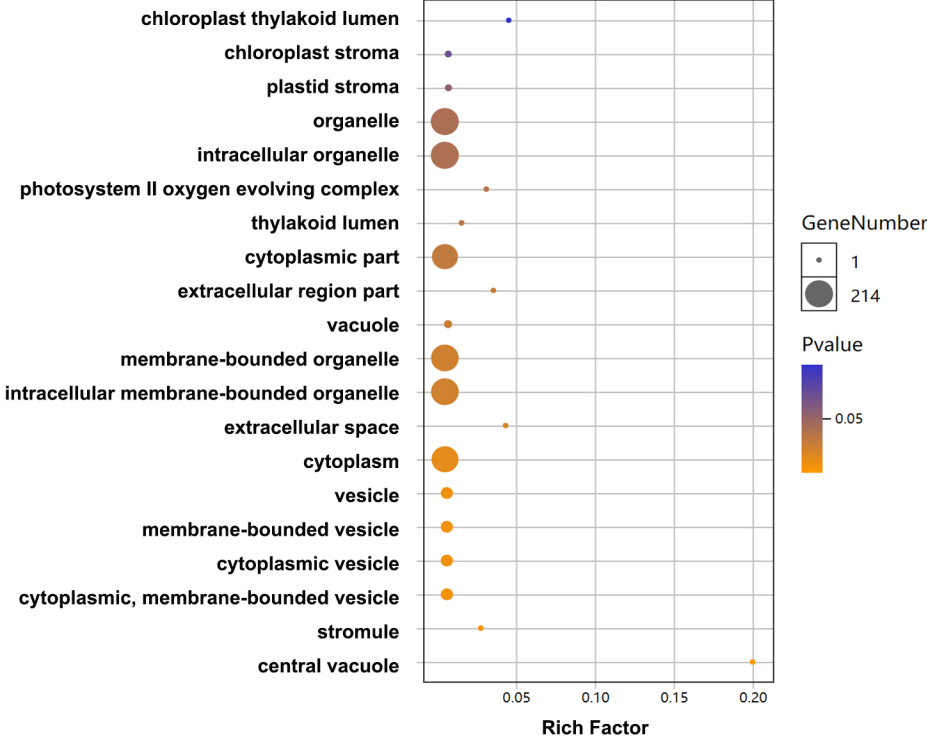

Supplement: Supplementary file 5 — Additional file 5: Figure S3. Functional classification of DEGs using GO analysis. The horizontal axis shows the enrichment factors of the GO terms. The vertical axis shows the top 20 enriched GO terms in the biological process, molecular function, and cellular component categories. The size of the circle indicates the number of genes enriched in each term. The color of the circle indicates the P value of the enriched term. [file 13068_2019_1534_MOESM5_ESM.pdf]
