# Supplementary material for: Comparative transcriptome analysis provides key insights into seedling development in switchgrass (Panicum virgatum L.)
Source: Biotechnol Biofuels. 2019 Aug 5;12:193. doi: 10.1186/s13068-019-1534-4 (PMC6683553; doi:10.1186/s13068-019-1534-4)
Supplement: Supplementary file 8 — Additional file 8: Figure S4. Homology analysis of switchgrass and maize leaf development. The left panel shows DEGs in switchgrass that are homologous to maize. Red color indicates upregulated expression, and green indicates downregulated expression in sd seedlings. The right panel shows gene IDs for maize. Orange and blue indicate positive and negative correlations, respectively, with leaf development in maize. [file 13068_2019_1534_MOESM8_ESM.pdf]

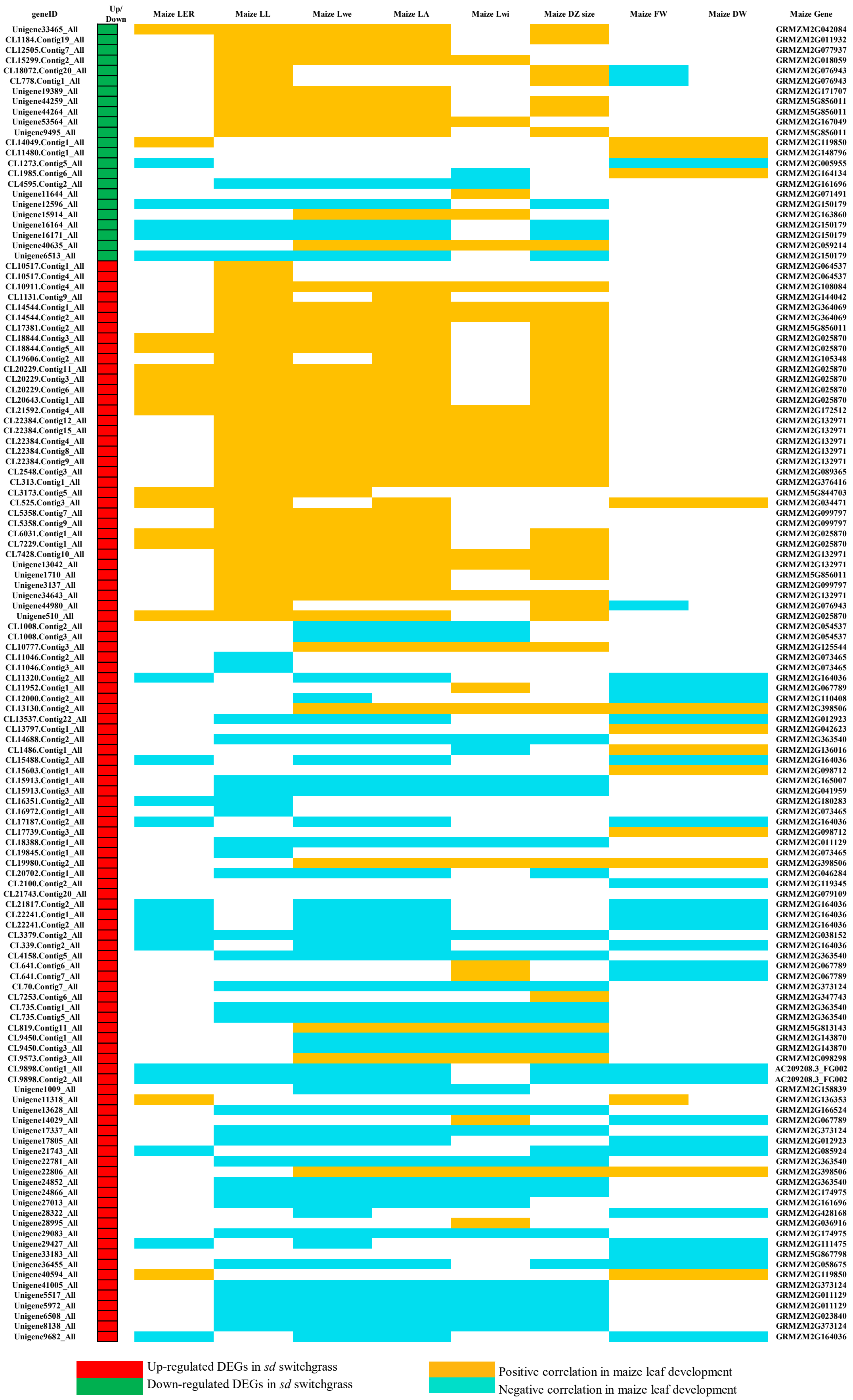

LER: leaf elongation rate; LL: leaf length; Lwe: leaf weight; LA: leaf area; Lwi: leaf width; DW: dry weight; DZ: division zone; FW: fresh weight
